# Supplementary material for: Bacopasides I and II Act in Synergy to Inhibit the Growth, Migration and Invasion of Breast Cancer Cell Lines
Source: Molecules. 2019 Sep 30;24(19):3539. doi: 10.3390/molecules24193539 (PMC6803832; doi:10.3390/molecules24193539)
Supplement: Supplementary file 1 [file molecules-24-03539-s001.pdf]

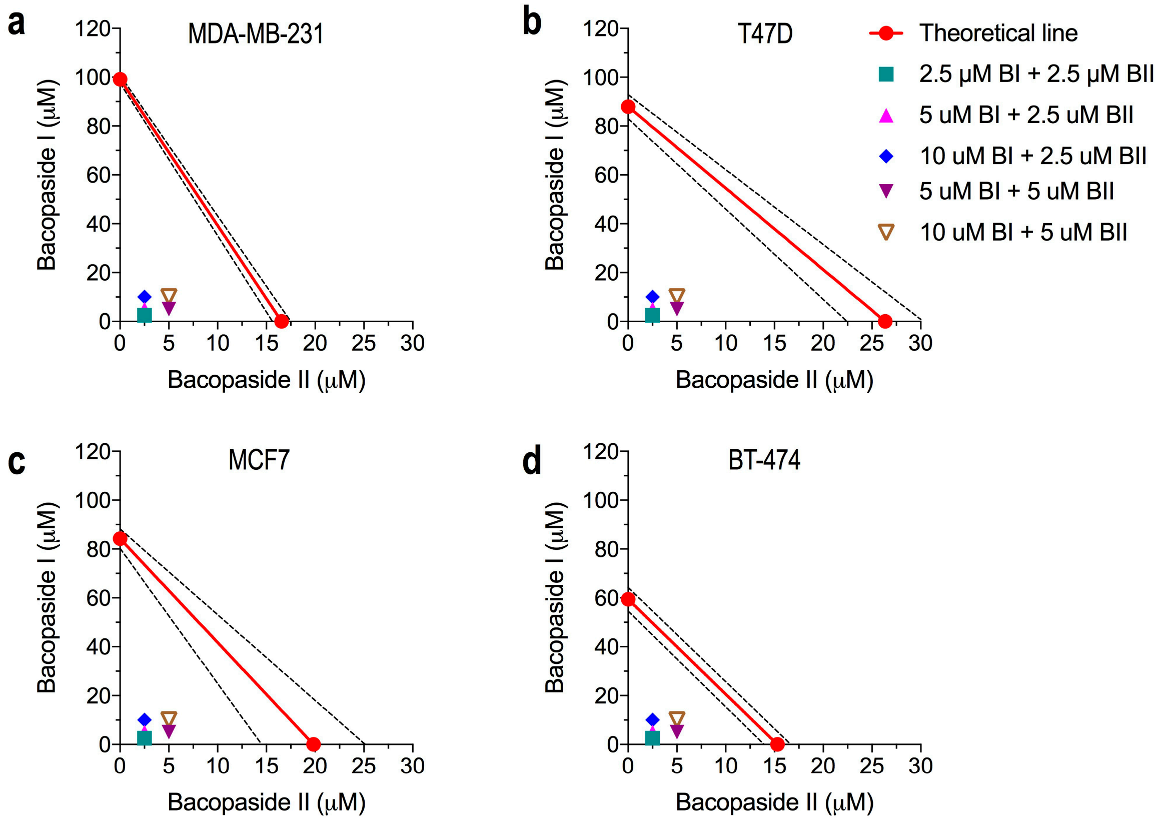

Figure S1. Synergistic combinations of bac I and II were determined by isobologram analysis and combination indices. IC50 values for bac I and bac II used in isolation were used to create an isobologram for MDA-MB-231 (**a**), T47D (**b**), MCF7 (**c**), and BT-474 (**d**). Different ratios (1:1; 2:1 and 4:1) of bac I and bac II at doses well below the IC50s for each drug are plotted. Dotted lines are upper and lower 95% confidence intervals.

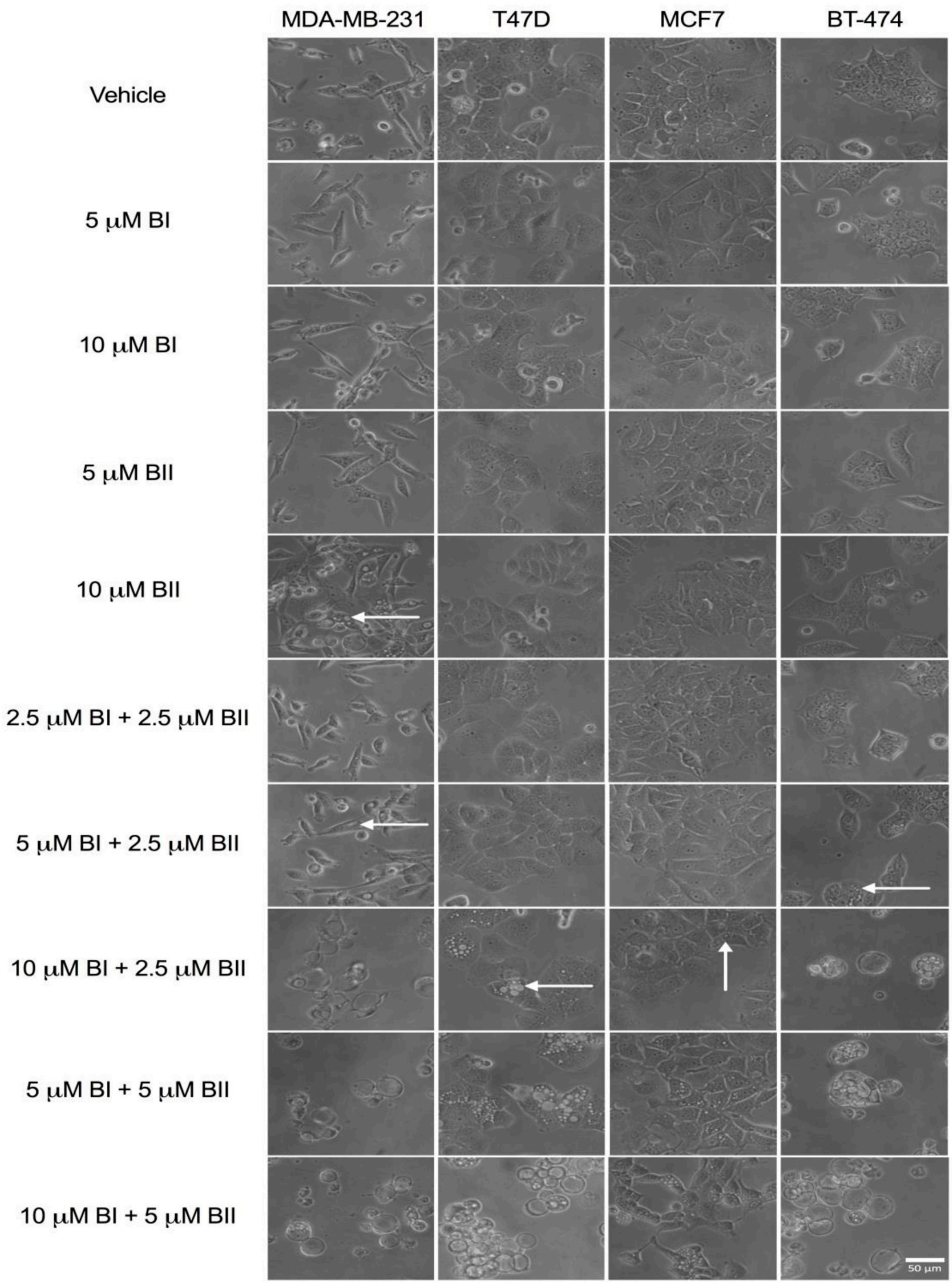

Figure S2. Bac I and II combined dose below the IC50s induced vacuoles and changes reminiscent of apoptosis. Images were captured using a Nikon Eclipse TS100 (Nikon, Tokyo, Japan). Morphological changes were more prominent when the doses were combined, compared to when used in isolation. Arrows indicate the lowest doses, singly and in combination, at which morphological changes became apparent for each cell line. Bac I and bac II are abbreviated to BI and BII respectively. Magnification 400x. Scale bar = 50 μm.

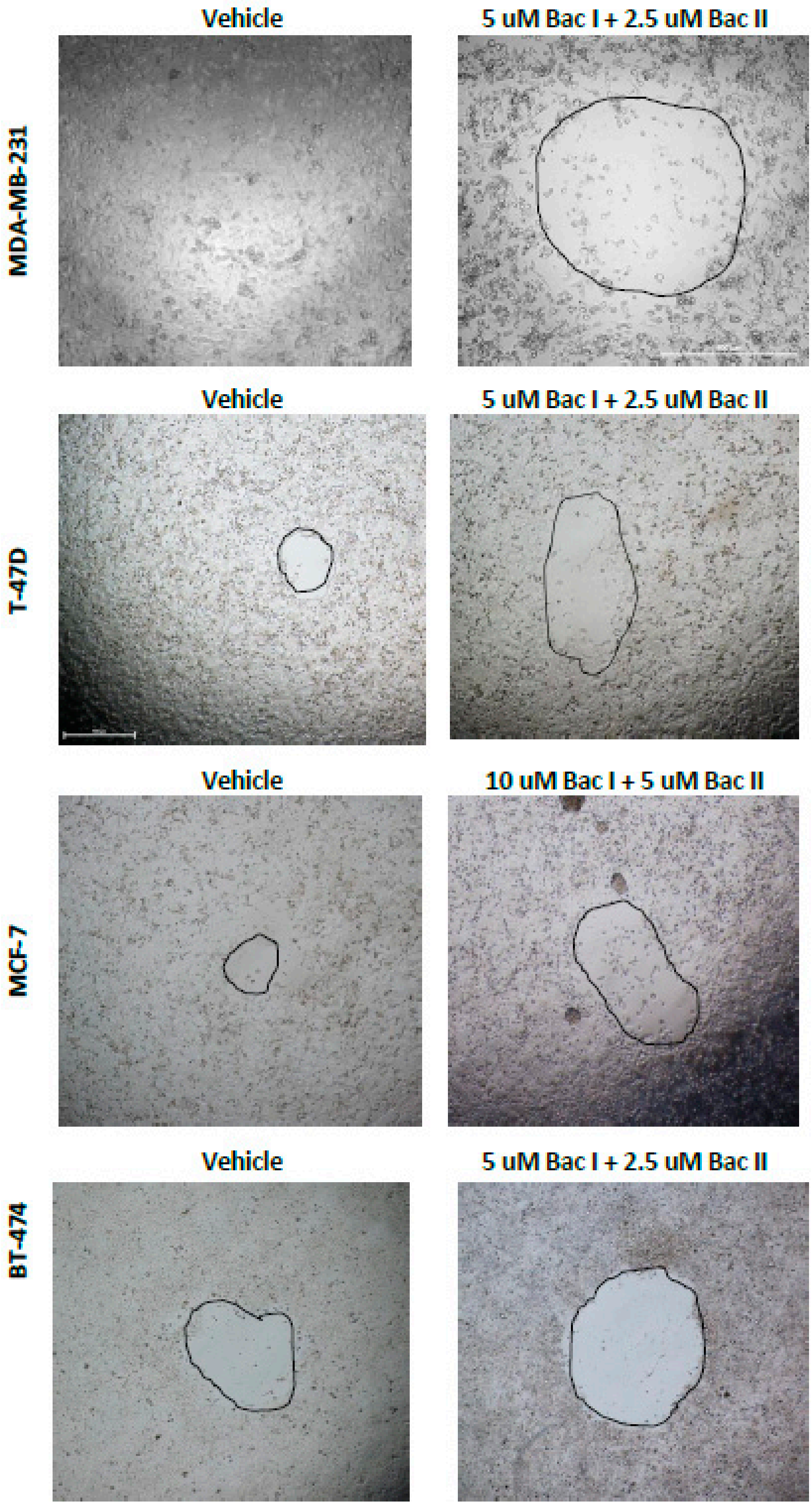

Figure S3. Migration (wound closure) was significantly inhibited by combined doses of Bac I and Bac II. The images of MDA-MB-231 were taken at 100 x magnification, other images at 40 x magnification. Scale bars = 500 μm.

Table S1. Combination indices for breast cancer cell lines.

	Cell line CIs*			
	**MDA-MB-231**	**T47D**	**MCF7**	**BT-474**
**Combination dose**				
2.5 µM BI + 2.5 µM BII	0.176 (0.168-0.186)	0.123 (0.109-0.141)	0.156 (0.128-0.203)	0.205 (0.189-0.225)
5 µM BI + 2.5 µM BII	0.201 (0.192-0.211)	0.152 (0.136-0.172)	0.185 (0.156-0.234)	0.247 (0.228-0.271)
10 µM BI + 2.5 µM BII	0.252 (0.242-0.262)	0.208 (0.190-0.232)	0.245 (0.213-0.297)	0.332 (0.305-0.362)
5 µM BI + 5 µM BII	0.352 (0.335-0.371)	0.246 (0.219-0.283)	0.311 (0.255-0.407)	0.411 (0.378-0.450)
10 µM BI + 5 µM BII	0.403 (0.385-0.422)	0.303 (0.273-0.343)	0.371 (0.312-0.469)	0.495 (0.455-0.542)

Combination indices (CIs) were calculated from the IC_{50}s and combination doses used in Figure S1 and are shown with upper and lower 95% confidence intervals in brackets. *CI < 1, synergistic; CI = 1, additive; CI > 1, antagonistic.
